# ARTIFICIAL INTELLIGENCE IN MENTAL HEALTH FOR THE AGING POPULATION: A SCOPING REVIEW

**DOI:** 10.1093/geroni/igad104.2598

**Published:** 2023-12-21

**Authors:** Duo Helen Wei, Bianca Hernandez, David Burdick

**Affiliations:** Stockton University, Galloway, New Jersey, United States; Rutgers University, Galloway, New Jersey, United States; Stockton University, Galloway, New Jersey, United States

## Abstract

Artificial intelligence (AI) has significant potential in mental health research and service delivery for the aging population. This poster provides an overview of its evolving utilization, including research methods, data sources, and predictors of mental health. The review of 26 articles from 2015 to 2023 found that surveys/questionnaires, clinical data, non-clinical data, and electronic health records are the most commonly used data sources, with clinical data being the most common, accounting for more than 50% of the articles. The frequently utilized AI techniques in mental health research for older adults were supervised learning (65.4%), deep learning (42.3%), unsupervised learning (7.7%), NLP (7.7%), and semi-supervised learning (3.8%). Mental health factors that received the most attention in the research were anxiety/depression (65.4%), dementia (Alzheimer’s) (38.5%), mild cognitive impairment (23.1%), suicidal ideation (15.4%), and trajectory of cognitive decline (7.7%). AI is currently employed in various aspects of mental health research for older adults, including identifying and diagnosing mental health conditions at an early stage, monitoring mental well-being, assessing the severity of mental health conditions, predicting cognitive trajectories, and providing assistance in treatment. The recent application of generative language models, such as ChatGPT, has further advanced the field of AI in mental health research. However, ethical concerns such as bias, privacy, and transparency must be addressed before widespread implementation. In conclusion, AI has great potential as well as risks and ethical concerns for mental health research and service delivery to aging populations, but further research is needed.

